# Neutrophil extracellular traps aggravate neuronal endoplasmic reticulum stress and apoptosis via TLR9 after traumatic brain injury

**DOI:** 10.1038/s41419-023-05898-7

**Published:** 2023-06-26

**Authors:** Liang Mi, Xiaobin Min, Mingming Shi, Liang liu, Yanfeng Zhang, Yanlin Zhu, Peng Li, Yan Chai, Fanglian Chen, Quanjun Deng, Shu Zhang, Jianning Zhang, Xin Chen

**Affiliations:** 1 Department of Neurosurgery, Tianjin Medical University General Hospital, Tianjin, P.R. China; 2 Tianjin Neurological Institute, Key Laboratory of Post-Trauma Neuro-Repair and Regeneration in Central Nervous System, Ministry of Education, Tianjin Key Laboratory of Injuries, Variations and Regeneration of Nervous System, Tianjin, P.R. China; 3 Department of Neurosurgery, Baodi Clinical College, Tianjin Medical University, Baodi, Tianjin, P.R. China

**Keywords:** Cell death in the nervous system, Endoplasmic reticulum

## Abstract

Endoplasmic reticulum (ER) stress and ER stress-mediated apoptosis play an important role during secondary brain damage after traumatic brain injury (TBI). Increased neutrophil extracellular traps (NETs) formation has been demonstrated to be associated with neurological damage after TBI. However, the correlation between ER stress and NETs remains unclear, and the specific function of NETs in neurons has not been defined. In this study, we found that the levels of NETs circulating biomarkers were remarkably elevated in the plasma of TBI patients. We then inhibited NETs formation by peptidylarginine deiminase 4 (PAD4, a critical enzyme for NETs formation) deficiency and discovered that ER stress activation and ER stress-mediated neuronal apoptosis were reduced. The degradation of NETs via DNase I showed similar outcomes. Furthermore, overexpression of PAD4 aggravated neuronal ER stress and ER stress-associated apoptosis, while TLR9 antagonist administration abrogated the damage caused by NETs. In addition to in vivo experiments, in vitro experiments revealed that treatment with a TLR9 antagonist alleviated NETs-induced ER stress and apoptosis in HT22 cells. Collectively, our results indicated that ER stress as well as the accompanying neuronal apoptosis can be ameliorated by disruption of NETs and that suppression of the TLR9-ER stress signaling pathway may contribute to positive outcomes after TBI.

## Introduction

Traumatic brain injury (TBI) plays a primary role in the occurrence of disability and mortality worldwide, resulting in heavy financial and physical burdens for the patients [1, 2]. Despite comprehensive basic science research and clinical studies on TBI for >100 years, few effective therapeutics or valid pharmacological treatments for TBI exist [3]. The pathogenic mechanism of TBI includes irreversible primary insult and multifactorial secondary brain injury, and secondary brain injury may last from hours after injury to even years [4], leading to neuronal death in areas beyond the site of impact [5]. While the primary impact is transient, secondary insult is an ideal opportunity for therapeutic treatments [6].

Neutrophils are viewed as the frontline of the innate immune system defense against bacteria [7]. In addition to their microbe-killing ability, neutrophils can also form neutrophil extracellular traps (NETs) [8]. NETs are a weblike network consisting of DNA fibers and intracellular proteins such as histones, myeloperoxidase (MPO), and other antimicrobial proteins [9]. Peptidylarginine deiminase 4 (PAD4) plays an important role in chromatin decondensation via the citrullination of histones, leading to the extrusion of NETs [10]NETs can encapsulate and kill pathogens such as viruses and bacteria [11], but it has been demonstrated that excessive formation of NETs is associated with a series of autoimmune diseases, such as thrombosis, inflammation, diabetes and cancer [12–14].In recent years, NETs in TBI model mice and TBI patients have also been observed to contribute to edema after impact [15] and have been found to function through signaling pathways such as Toll-like receptor 4 (TLR4)-dependent mechanisms [16].

The endoplasmic reticulum (ER) is a key cellular organelle in which protein synthesis, maturation, folding, modification and degeneration occur [17]. When the balance of protein folding is disturbed, ER stress occurs, which can trigger neuronal apoptosis [18]. It has been demonstrated that TLR2 stimulation to activate ER stress and subsequent NETs formation may protect smooth muscle cell-rich plaques that are susceptible to superficial erosion and thrombotic complications [19]. The roles of individual ER stress and NETs formation disorders have been comprehensively explored in TBI [20], but the correlations between these disorders and their roles in TBI remain unclear.

The relationship between PAD4 and ER stress activation has been verified in several studies. In a pristane-induced diffuse alveolar hemorrhage mouse model, researchers discovered that deletion of PAD4 in myeloid cells could inhibit lung inflammation and IFN-driven responses as well as ER stress stimulation [21]. In another sepsis-induced intestinal injury model, PAD4 deficiency was found to decrease ER stress activation [22]. Both studies indicated that PAD4 is not only important for NETs formation but also for ER stress activation. However, the interaction between PAD4 and ER stress in a TBI model has not been described.

In this study, we explored the influence of NETs regulation on ER stress in neurons after TBI and subsequent ER stress-mediated apoptosis. Our research confirmed that NETs biomarker levels were significantly increased in TBI patients. Furthermore, NETs depletion via both PAD4 deficiency and DNase I alleviated neuronal ER stress in TBI model mice and improved neurological outcomes. Remarkably, we demonstrated that NETs-induced neuronal apoptosis could be regulated by ER stress via Toll-like receptor 9 (TLR9), and our in vitro experiments produced similar results.

## Results

### TBI patients have increased NETs plasma levels

**Fig. 1:**
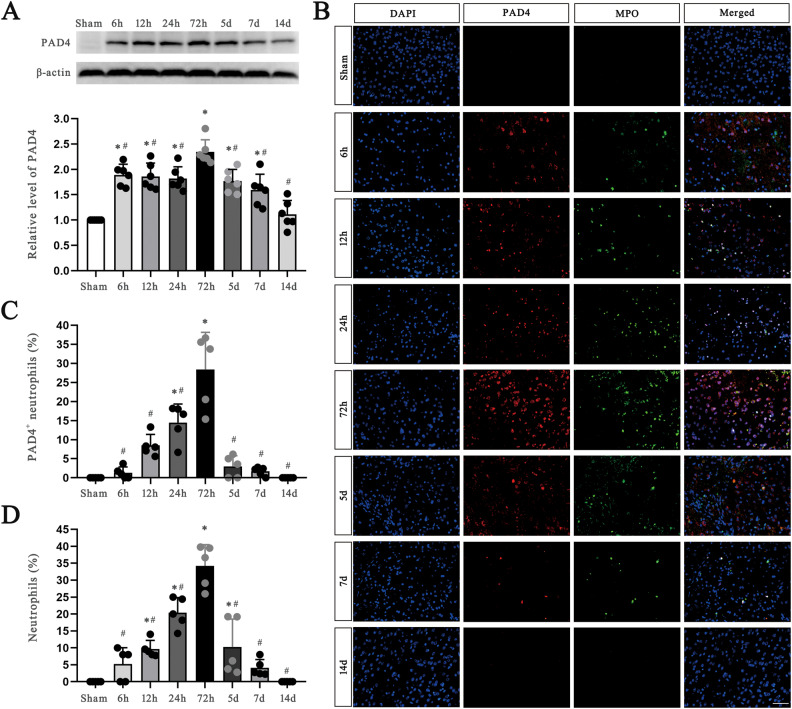
Dynamic expression of PAD4 and neutrophils after TBI over a period of time. **A** Protein levels of PAD4 in the ipsilateral cortex of mice from the sham group and TBI group at 6 h, 12 h, 24 h, 72 h, 5 days, 7 days and 14 days (*n* = 6). **B**–**D** Representative photographs of immunofluorescence staining of PAD4 with MPO, a neutrophil biomarker, and quantification of the percentage of and PAD4^+^MPO^+^ cells and MPO^+^ cells among total cells in the pericontusional cortex of mice from the sham group and TBI group at 6 h, 12 h, 24 h, 72 h, 5 days, 7 days and 14 days (*n* = 5). Nuclei were stained with DAPI. Scale bar = 50 µm. PAD4 peptidylarginine deiminase 4, TBI traumatic brain injury, MPO myeloperoxidase. Data are expressed as the mean ± SD. *p < 0.05 compared with sham, #p < 0.05 compared with 72 h.

To investigate whether NET levels are different in the plasma of TBI patients, we first detected the levels of circulating cell-free DNA (cf.-DNA), a biomarker of plasma NETs, and found that they were significantly elevated in TBI patients compared with healthy controls. We also found that the level of the NET biomarker CitH3 was significantly increased in neutrophils from blood of TBI patients compared to those of healthy controls (Supplementary Fig. 1A, B). (The figure of full lengths western blot is provided as supplemental material named Full lengths western blots in Fig. 1).

### Dynamic expression of PAD4 and neutrophils after TBI over a period of time

PAD4 is a critical enzyme for NETs formation; thus, we designed a 14-day experiment to observe the time point in which the expression level of PAD4 was highest after TBI. The western blotting results showed that the relative level of PAD4 began to increase at 6 h, peaked at 72 h, and returned to slightly above baseline at 14 d post injury, and immunofluorescence staining showed a similar result of PAD4^+^ neutrophils increasing after insult and peaking at 72 h. (Fig. 1A-C). The full length western blot results are provided in Supplementary Fig. 1.

In addition, immunofluorescence staining also showed that there was an increase in the percentage of neutrophils in the pericontusional region of the brain. The percentage of neutrophils increased at 12 h after TBI, peaked at 72 h, and then returned to baseline within 7 days. (Fig. 1B, D). Considering the similar time points of NET formation and neutrophil infiltration, we chose 72 h as the time point for sacrifice of the TBI mice in the following experiments unless otherwise specified.

### Inhibition of NETs formation reduces ER stress activation and apoptosis after TBI

**Fig. 2:**
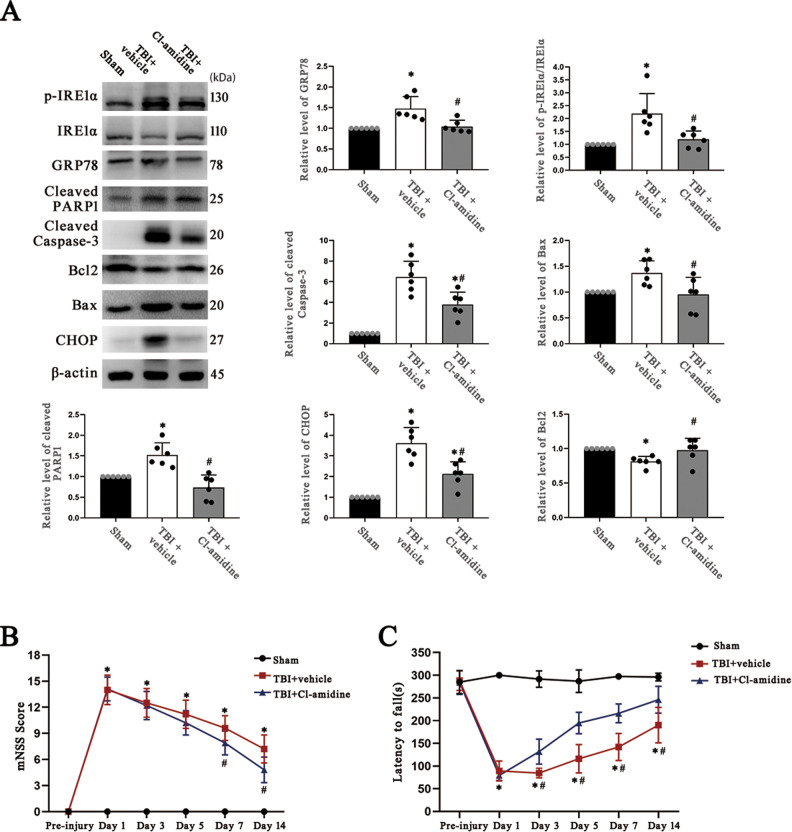
PAD4 inhibitor (Cl-amidine) treatment reduces ER stress and apoptosis and improves neurological outcomes after TBI. **A** Protein levels of ER stress biomarkers (p-IRE1α/IRE1α and GRP78) and apoptotic signaling molecules (cleaved PARP1, cleaved Caspase-3, CHOP, Bcl2/Bax) in the ipsilateral cerebral cortex of mice at 72 h after TBI were assessed by western blotting. The intensity was quantified by ImageJ software (*n* = 6). **B** The modified neurological severity (mNSS) scores of mice during a 14-day follow-up period after TBI (*n* = 10). **C** The latency to fall in the rotarod test during the 14-day follow-up period post TBI in mice (*n* = 10). PAD4 peptidylarginine deiminase 4, ER endoplasmic reticulum, TBI traumatic brain injury. Data are expressed as the mean ± SD. *p < 0.05 compared with sham, #*p* < 0.05 compared with TBI + Cl-amidine.

We used the PAD4 inhibitor Cl-amidine to disrupt the formation of NETs and explore the role of NETs formation in ER stress activation. Western blot analysis indicated that the expression of the ER stress molecular markers p-IRE1α/IRE1α and GRP78 and the proapoptotic molecular markers CHOP, cleaved caspase-3, and cleaved PARP-1 was significantly upregulated and that the expression of the antiapoptotic molecular marker Bcl2/Bax was markedly downregulated in the cortex after TBI in mice. However, treatment with Cl-amidine reversed these effects (Fig. 2A). The full length western blot results are provided in Supplementary Fig. 2.

**Fig. 3:**
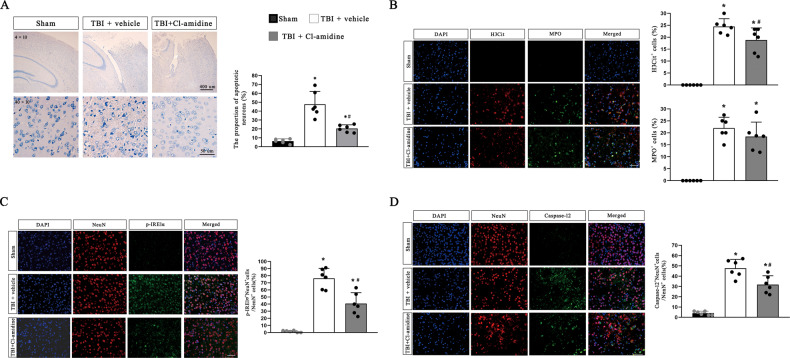
Administration of a PAD4 inhibitor (Cl-amidine) reduces NETS formation, ER stress in neurons and ER stress-mediated neuronal apoptosis after TBI. **A** Representative images of Nissl staining and quantitative analyses of the proportion of apoptotic neurons (%) in the ipsilateral cerebral cortex of mice at 72 h after TBI (*n* = 6). Scale bar on the top = 400 µm, scale bar on the bottom = 50 µm. **B** Representative immunofluorescence images of H3Cit^+^ (NETS biomarker) and MPO^+^ (neutrophil biomarker) cells, and quantification of the percentage of H3Cit^+^ and MPO^+^ cells at 72 h after TBI (*n* = 6). Nuclei were stained with DAPI. Scale bar = 50 µm. **C**, **D** Representative immunofluorescence images of NeuN (neuron marker), p-IRE1α (an ER stress biomarker), and Caspase-12 (an ER stress-mediated apoptosis biomarker) and quantification of the percentage of p-IRE1α^+^NeuN^+^ cells and caspase-12^+^ NeuN^+^ cells among total NeuN^+^ cells in the pericontusional cortex of mice at 72 h after TBI (*n* = 6). Nuclei were stained with DAPI. Scale bar = 50 µm. PAD4 peptidylarginine deiminase 4, ER endoplasmic reticulum, TBI traumatic brain injury, MPO myeloperoxidase, H3Cit citrullinated histone 3. Data are expressed as the mean ± SD. *p < 0.05 compared with sham, #*p* < 0.05 compared with TBI.

The Nissl staining results demonstrated that there was a large proportion of apoptotic neurons in the pericontusional region after TBI, whereas the proportion was decreased after Cl-amidine administration (Fig. 3A). The immunofluorescence staining results confirmed this discovery. The proportion of neurons positive for p-IRE1α (an ER stress marker) and Caspase-12 (an ER stress-mediated apoptosis marker) was significantly reduced in the injured cortex in TBI model mice treated with Cl-amidine compared to those treated with DMSO solution (Fig. 3C, D), and the administration of Cl-amidine also decreased the formation of NETs compared with that in the TBI group, while the expression of neutrophil markers in the pericontusional region was not changed by Cl-amidine treatment (Fig. 3B).

In addition, the modified neurological severity scores (mNSS) and rotarod test results showed that Cl-amidine-treated TBI model mice exhibited improved neurological outcomes (Fig. 2B, C). These data suggest that inhibition of NETs formation can alleviate neuronal ER stress activation and ER stress-mediated apoptosis and improve neurological outcomes.

### Degradation of NETs reduces ER stress activation and apoptosis after TBI

**Fig. 4:**
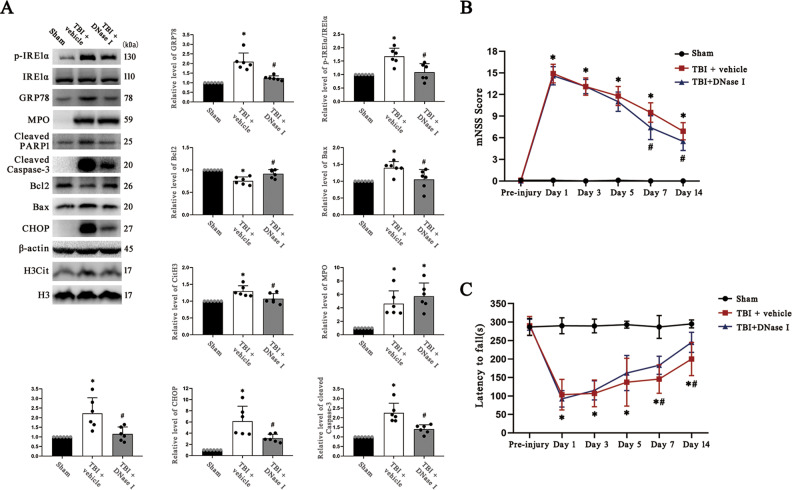
DNase 1 inhibits neutrophil infiltration, NETs existence, ER stress and apoptosis and ameliorates neuronal function and motor coordination deficits after TBI. **A** Protein levels of neutrophils (MPO), NETs (H3Cit), ER stress biomarkers (p-IRE1α/IRE1α and GRP78), and apoptotic signaling molecules (cleaved PARP1, cleaved Caspase-3, CHOP, Bcl2/Bax) in the pericontusional cortex of mice at 72 h after TBI were assessed by western blotting. The intensity was quantified using ImageJ software (*n* = 6). **B** The modified neurological severity (mNSS) scores of mice during a 14-day follow-up period post TBI (*n* = 6). **C** The latency to fall in the rotarod test during the 14-day follow-up period after TBI in mice (*n* = 6). MPO myeloperoxidase, H3Cit citrullinated histone 3, ER endoplasmic reticulum, TBI traumatic brain injury. Data are expressed as the mean ± SD. *p < 0.05 compared with sham, #*p* < 0.05 compared with TBI.

We used DNase I to degrade NETs and observe whether the degradation of NETs has an impact on ER stress. The western blotting results showed that DNase I treatment significantly suppressed ER stress activation and inhibited apoptosis in the pericontusional region after TBI as well as decreased the level of NETs, but the treatment did not change the level of neutrophil infiltration in the pericontusional region. (Fig. 4A). The full length western blot results are provided in Supplementary Fig. 4.

**Fig. 5:**
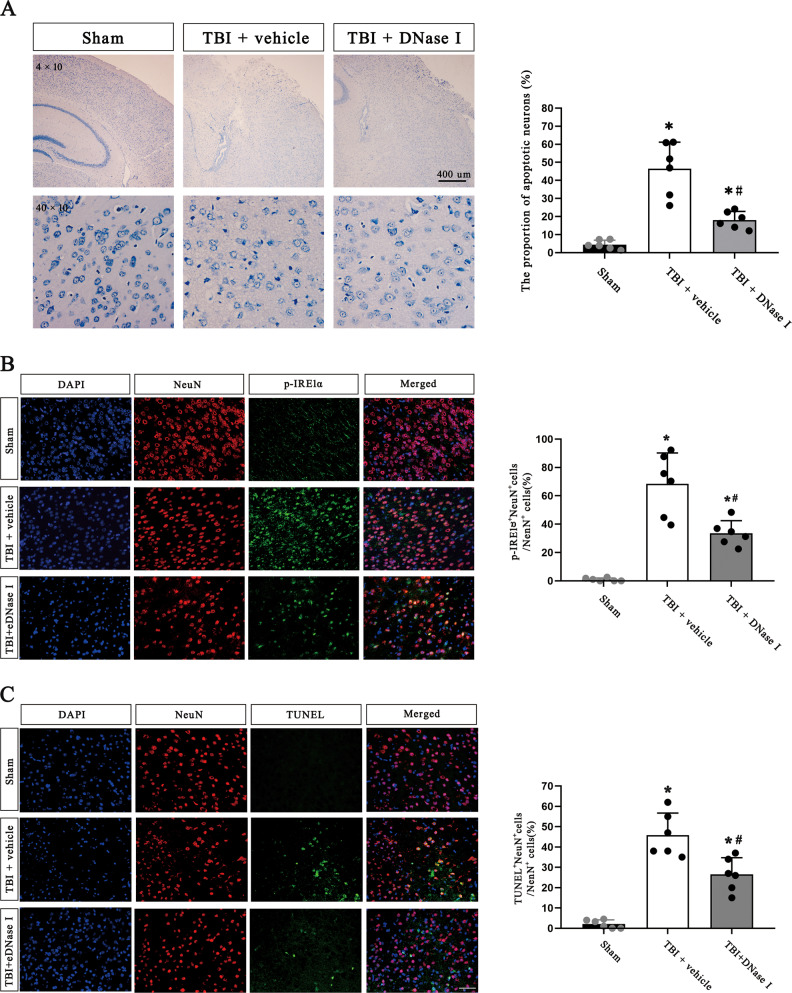
DNase 1 treatment alleviates ER stress in neurons and ER stress-mediated neuronal apoptosis after TBI. **A** Representative photographs of Nissl staining and quantitative analyses of the proportion of apoptotic neurons (%) in the pericontusional cortex of mice at 72 h after TBI (*n* = 6). Scale bar on the top = 400 µm, scale bar on the bottom = 50 µm. **B, C** Representative immunofluorescence images of NeuN (neurons), p-IRE1α (an ER stress biomarker) and TUNEL (an apoptosis biomarker), and quantification of the percentage of p-IRE1α^+^NeuN^+^ cells and TUNEL^+^ NeuN^+^ cells among total NeuN^+^ cells in the ipsilateral cerebral cortex of mice at 72 h after TBI (*n* = 6). Nuclei were stained with DAPI. Scale bar = 50 µm. ER endoplasmic reticulum, TBI traumatic brain injury, TUNEL (TdT)-mediated dUTP nick end labeling. Data are expressed as the mean ± SD. *p < 0.05 compared with sham, #*p* < 0.05 compared with TBI.

The Nissl staining results showed that there was an increased proportion of apoptotic neurons and increased shrinkage of neurons in the injured cortex after TBI, whereas the proportion of apoptotic neurons decreased and the morphology of neurons tended to be more typical in TBI model mice treated with DNase I (Fig. 5A). The immunofluorescence staining analysis indicated that compared to control mice, TBI model mice subjected to DNase I injection had a decreased proportion of p-IRE1α-positive neurons and TUNEL-positive neurons in the pericontusional region (Fig. 5B, C).

Furthermore, administration of DNase I improved the mNSSs and increased the latency to fall in the rotarod test of TBI model mice (Fig. 4B, C). These results indicated that NETs degradation inhibits ER stress and apoptosis, improves neurological outcomes and ameliorates motor coordination deficits.

### TLR9 mediates NETs-induced ER stress activation and apoptosis

To investigate the role TLR9 plays in ER stress activation, the TLR9 antagonist OND-2088 was used. Subsequently, to further identify the role of NETs formation in TBI, we used a PAD4 adenovirus to overexpress PAD4.

**Fig. 6:**
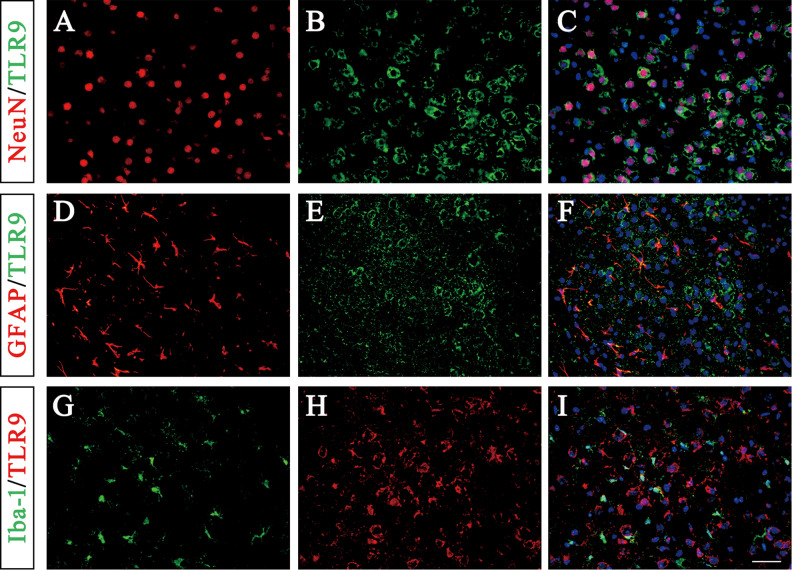
TLR9 immunofluorescence in the pericontusional cortex of TBI model mice. **A**–**C** Colocalization of TLR9 (green-B) with neurons stained with NeuN (red-A). **D**–**F** Colocalization of TLR9 (green-E) with astrocytes stained with GFAP (red-D). **G**–**I** Colocalization of TLR9 (red-G) with microglia stained with Iba-1 (red-A). The nuclei were stained with DAPI. Scale bar = 50 µm. TLR9 Toll-like receptor 9, TBI traumatic brain injury.

The colocalization analysis demonstrated that TLR9 was present mainly in neurons according to immunofluorescence staining for the neuronal marker NeuN, whereas relatively less positive staining for TLR9 was observed in astrocytes (identified by a GFAP antibody) and microglia (identified by Iba-1), indicating that TLR9 is primarily expressed in neurons in the pericontusional region in TBI model mice (Fig. 6).

**Fig. 7:**
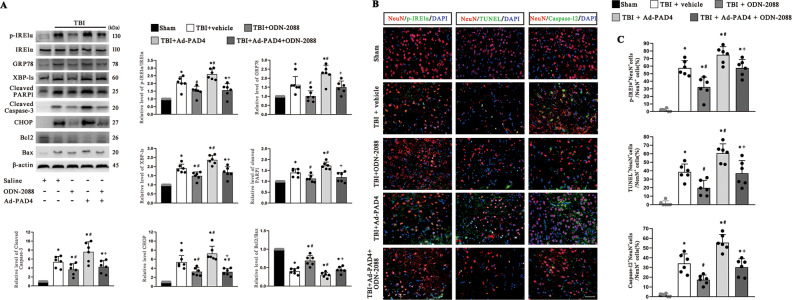
TLR9 antagonist inhibits neuronal ER stress and apoptosis and reverses the impact induced by PAD4 overexpression. **A** Western blotting bands of ER stress biomarkers (p-IRE1α/IRE1α, GRP78 and XBP-1s) and apoptotic signaling molecules (cleaved PARP1, cleaved Caspase-3, CHOP, Bcl2/Bax) in the ipsilateral cerebral cortex of mice at 72 h after TBI. The intensity was quantified using ImageJ software (*n* = 6). **B** Representative immunofluorescence images of NeuN (neurons), p-IRE1α (an ER stress biomarker), TUNEL (an apoptosis biomarker) and Caspase-12 (an ER stress-mediated apoptosis biomarker) in the pericontusional cortex after TBI. Nuclei were stained with DAPI. Scale bar = 50 µm. **C** Quantitative analyses of the percentage of p-IRE1α^+^NeuN^+^ cells, TUNEL^+^NeuN^+^ cells and caspase-12^+^ NeuN^+^ cells among total NeuN^+^ cells (*n* = 6). PAD4 peptidylarginine deiminase 4, ER endoplasmic reticulum, TLR9 Toll-like receptor 9, TBI traumatic brain injury, TUNEL (TdT)-mediated dUTP nick end labeling. Data are expressed as the mean ± SD. *p < 0.05 compared with sham, #*p* < 0.05 compared with TBI, +*p* < 0.05 compared with TBI + Ad-PAD4.

The western blot results demonstrated that ODN-2088 administration alleviated ER stress activation and apoptosis, while overexpression of PAD4 markedly upregulated the expression of the ER stress molecular markers p-IRE1α/IRE1α, XBP-1s and GRP78 and the proapoptotic molecular markers CHOP, cleaved caspase-3, and cleaved PARP-1 and downregulated the expression of antiapoptotic molecular markers Bcl2/Bax compared with that in TBI model mice injected with the control adenovirus. However, the effect of PAD4 overexpression could be reversed by ODN-0288 administration (Fig. 7A). The full length western blot results are provided in Supplementary Fig. 7.

Immunofluorescence staining showed that the proportions of p-IRE1α-positive (ER stress marker) neurons, TUNEL-positive (apoptosis marker) neurons and caspase-12-positive (ER stress-mediated apoptosis marker) neurons were significantly reduced after treatment with ODN-2088. While the levels of these markers were remarkably elevated via PAD4 overexpression compared to those in TBI model mice treated with control adenovirus, the effects of PAD4 overexpression were reversed by administration of ODN-2088 (Fig. 7B, C). These results demonstrated that TLR9 may contribute to NETs-induced ER stress activation and ER stress-mediated apoptosis in neurons after TBI.

### TLR9 mediates NETs-induced ER stress activation in HT22 cells

**Fig. 8:**
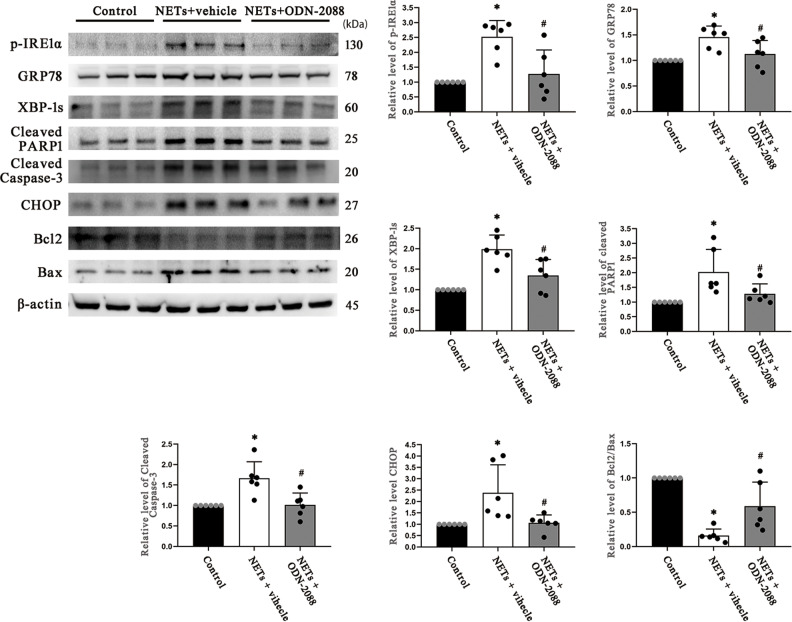
TRL9 antagonist alleviates ER stress and apoptosis resulting from NET administration in HT22 neurons. Western blotting bands of ER stress biomarkers (p-IRE1α/IRE1α, GRP78 and XBP-1s) and apoptotic signaling molecules (cleaved PARP1, Caspase-3, CHOP, Bcl2/Bax) in HT22 neurons. The intensity was quantified using ImageJ software (*n* = 6). TLR9 Toll-like receptor 9, ER endoplasmic reticulum, PAD4 peptidylarginine deiminase 4 Data are expressed as the mean ± SD. *p < 0.05 compared with control, #*p* < 0.05 compared with NETs.

HT22 neurons were cocultured with NETs to explore the role that NETs play in ER stress activation. From the western blotting results, we observed that HT22 cells cocultured with NETs expressed high levels of ER stress molecular markers and apoptosis molecular markers, while treatment with ODN-2088 reversed the effect of NETs (Fig. 8). These data demonstrated that NETs can directly stimulate ER stress and that TLR9 plays an important role in NETs-induced ER stress activation and apoptosis in HT22 cells. The full-length western blot results are provided in Supplementary Fig. 8.

## Discussion

In the current study, we found increased plasma NETs levels in TBI patients. Furthermore, NETs formation interference and NET degradation had inhibitory effects on ER stress activation and apoptosis in the neurons of TBI model mice. Another novel discovery was that a TLR9 antagonist helped reduce ER stress and ER stress-mediated apoptosis in TBI model mice and could also reverse the activation of ER stress resulting from PAD4 overexpression.

Clinical results have shown that neutrophil circulating levels increase by 4-fold and these neutrophils infiltrate the cortical and subcortical parenchyma over several days after TBI [23, 24]. NETs generated from activated neutrophils damage the central nervous system (CNS) through many mechanisms after TBI, such as the disruption of brain cells and vascular endothelial cells [25–27]. Considering the terrible role of ER stress activation in the process of secondary brain injury [15] we hypothesized that there was a connection between NETs and ER stress in TBI. Circulating NETs biomarkers have been observed in TBI patients, and similar results were obtained in our research; NETs levels were increased in the plasma of TBI patients. Considering our previous study, which showed that ER stress and apoptosis were elevated in TBI model mice [18], we hypothesized that a connection between NETs infiltration and ER stress activation exists, implicating a potential target for the treatment of TBI.

PAD4 is an enzyme expressed in peripheral blood neutrophils that can catalyze histone citrullination, leading to the decondensation of chromatin, which is critical for NETs formation [10]. To determine the effect of our treatments, which mainly modulated the formation and existence of NETs, we first identified the time point when the expression of PAD4 was highest after TBI and found that PAD4 expression in neutrophils peaked at 72 h after TBI. Consistent with our previous study, which showed that the expression levels of apoptotic markers peaked at 72 h after TBI [18], we speculated that 72 h was the best time to investigate the effects of our treatments in TBI model mice. Therefore, we sacrificed the mice 72 h after TBI to investigate the changes in molecular markers and examine the therapeutic effects of the treatments.

IRE1α is a transmembrane protein attached to the ER that plays a crucial role in regulating the unfolded protein response (UPR) [28], which activates ER stress and induces apoptosis under stress such as TBI [29]. In addition, activation of IRE1α has recently been observed to be associated with NETs formation [30], so we chose p-IRE1α, the activated form of IRE1α, as the molecular marker of ER stress activation. Compared to TBI group mice, the TBI model mice treated with the PAD4 inhibitor Cl-amidine showed reductions in NETs levels, ER stress activation, and ER stress-mediated apoptosis in neurons, and the results of the mNSS and rotarod test also showed obvious progress during a 14-day follow-up period after TBI, indicating that NETs play an important role in neuronal ER stress and that inhibition of NETs formation reduces ER stress in neurons and neuronal apoptosis as well as improves neurological outcomes after TBI.

Similar results were obtained when TBI mice were subjected to DNase I treatment. DNase I is an endogenous NET-degrading enzyme, and it has been demonstrated that circulating NETs in patients with severe neurotrauma are associated with suppression of serum DNase I activity [31]. DNase I has been found to degrade NETs and improve neurological function [15], which was also confirmed in our research. We are the first to discover that DNase I can reduce neuronal apoptosis via inhibition of ER stress.

However, neither Cl-amidine treatment nor DNase I treatment had an impact on the infiltration of neutrophils, which can be interpreted as both drugs exerting their influence by disrupting the formation and existence of NETs but not the infiltration of neutrophils. The activation of neutrophils is the cause of NETs release; thus, interference with NETs will not affect the level of neutrophils.

It has been proven that TLR9 plays a crucial role in recognizing conserved molecular structures and initiating downstream signaling pathways to mediate the immune response through pattern recognition receptors (PRRs) [32], and there is emerging evidence that TLRs are pivotal targets in mediating TBI [33]. Administration of the TLR9 antagonist ODN-2088 has been demonstrated to be an ideal treatment for several diseases, such as spinal cord injury, cerebral ischemia/reperfusion injury, liver injury and sepsis [22, 34–36]. While the effect of ODN-2088 on TBI remains unreported, based on previous observations, we focused on the impact of TLR9 antagonist treatment on TBI via ER stress signaling.

Surprisingly, we discovered that administration of a TLR9 antagonist inhibited ER stress after TBI and reduced ER-induced apoptosis in neurons. To further study the role of TLR9 inhibition in the NETs-induced neuronal dysfunction after TBI, we overexpressed PAD4, which has been observed to be markedly upregulated after TBI in a previous experiment, to increase the formation of NETs. Overexpression of PAD4 resulted in amplified ER stress activation and neuronal apoptosis. Consistent with the previous finding that inhibition of PAD4 alleviates ER stress activation and neuronal apoptosis after TBI, our findings reveal that targeting PAD4 is a promising strategy to alleviate ER stress. In addition, we also found that ODN-2088 treatment notably reversed the elevated ER stress caused by increased NETs formation, indicating the critical role of TLR9 antagonists in ameliorating NETs-induced neuronal ER stress after TBI.

To validate the effect of ODN-2088 on NETs in vitro, we cocultured HT22 neurons with NETs and found that administration of NETs stimulated ER stress and induced apoptosis of HT22 cells, and this phenomenon was reversed by treatment with ODN-2088. The results indicated that the TLR9 antagonist reversed ER stress resulting from NETs intervention in vitro. However, the mechanism by which ODN-2088 reverses the NET-induced effects in vitro needs further exploration.

Considering that NETs consist of neutrophil-derived DNA fibers and proteins [37], and that TLR9 is a potential DNA sensor [38], it is reasonable that TLR9 can regulate pathological reactions in response to NETs. In a study conducted by Jiang, TLR9 was found to be directly upregulated by freshly formed NETs, leading to stimulation of the NFκB, MAPK, and STAT3 pathways in tumor cells [39]. In addition, in a study focusing on intestinal barrier functions in sepsis, the NETs–TLR9–ER stress–reactive oxygen species (ROS) signaling pathway was proposed, and inhibition of TLR9 was demonstrated to be beneficial for mice suffering from gut barrier dysfunction by reducing ER stress activation and epithelial barrier disruption [22]. It has also been reported in a hepatic ischemia/reperfusion injury model that blocking TLR9 can downregulate the expression of PAD4 and Rac2, which are essential for NETs formation [40].

Notably, there were several limitations of the present study. First, we only observed the effect of targeting NETs on ER stress and apoptosis in neurons; we did not explore the effect on other types of brain cells, such as astrocytes and microglial cells. In addition to ER stress, several other important mechanisms in the CNS, such as neuroinflammation activation, blood–brain barrier disruption and autophagy activation, remain to be studied in the context of NETs. In addition, we did not identify a certain pathway through which TLR9 is activated by NETs. Finally, we investigated only the effects of TLR9 antagonism on TBI; the influence of TLR9 agonism needs to be observed as well.

In conclusion, our research demonstrated that NETs are involved in TBI-induced ER stress and apoptosis in neurons. Inhibiting NETs formation and degrading NETs both alleviates ER stress and ER stress-mediated apoptosis in neurons and improves the neurological outcomes of mice after TBI. Furthermore, we report for the first time that a TLR9 antagonist inhibits neuronal ER stress and apoptosis mediated by NETs in a mouse model of TBI. These findings indicated that suppression of the NETs-ER stress-TLR9 signaling pathway may be a promising strategy for TBI therapy.

## Methods

### Quantification of plasma DNA levels

Plasma was separated from whole blood by centrifugation at 150 × g for 15 min. Plasma DNA levels were quantified using the Quant-iT PicoGreen dsDNA Assay Kit (Invitrogen) according to the manufacturer’s instructions. Neutrophils were isolated from the blood using the Whole Blood Neutrophil Isolation Kit (Miltenyi Biotec) according to the manufacturer’s instructions. Human blood was obtained using a protocol that was reviewed and approved by the ethics committee of Tianjin Medical University General Hospital (IRB2022-WZ-125), and written informed consent was obtained from all patients and healthy controls.

### Animals

Adult male C57BL/6 mice weighing 20–25 g (8–10 weeks) at the time of surgery were purchased from the Experimental Animal Laboratories of the Academy of Military Medical Sciences (Beijing, China). They were individually housed in an enclosure with a controlled temperature of 18–22 °C, 55–60% humidity and a standard 12-h light/dark cycle (7:00 a.m. to 7:00 p.m.). The mice had access to food and water *ad libitum*. The mice were randomized into different groups with 5 or 6 mice in each group for most experiments, as for neurological behavioral test, the sample size in each group is 10 to ensure the relative uniformity in injury severity and to compare neurological impairments among mice receiving different treatments, all sample sizes were estimated prior to selection. The number of sacrificed mice and their suffering were limited to the lowest. All experimental procedures were conducted in strict accordance with the National Institutes of Health (NIH)’s “Guide for the Care and Use of Laboratory Animals” and approved by the Tianjin Medical University Animal Care and Use Committee (IRB2022-DW-55). The data were collected by investigators who were blinded to the experimental design in all experiments conditions and treatments.

### Mouse model of TBI

Mice were subjected to injury induced by controlled cortical impact (CCI), which has been widely described and broadly used as a preclinical model of head injury [41–43]. Briefly, mice was anesthetized by 10% chloral hydrate accompanied by morphine as a presurgery analgesic and placed in a stereotaxic apparatus. A 4.0-mm hole was drilled through the right parietal bone midway between bregma and lambda in the parietal bone centered 2 mm lateral from the sagittal suture with the dura matter intact. CCI was performed by a digital electromagnetic CCI device (eCCI Model 6.3; Custom Design, Richmond, VA) at a depth of 2.5 mm and a velocity of 5 m/sec over a 200 m/sec dwell time. The incision was closed immediately following injury, and the mice were placed in heated cages until they recovered from anesthesia at room temperature (RT).

### Drug administration

A stock solution of the PAD4 inhibitor Cl-amidine (506282, Millipore) was dissolved in dimethyl sulfoxide (DMSO) (Sigma‒Aldrich). The stock solution was dissolved in saline (5% v/v) and injected intraperitoneally (i.p.) into mice at a dose of 50 mg/kg 10 min after TBI and then every day until they were sacrificed.

Mice were injected intravenously (i.v.) with 10 mg/kg DNase I (deoxyribonuclease 1 human recombinant; enz-319-10000IU, ProSpec, Israel) 24 h after TBI and then 10 mg/kg i.v. every 12 h until sacrifice on day 3.

One hour prior to surgery, mice were intraperitoneally injected with the TLR9 antagonist ODN-2088 (10 μg/25 g body weight, tlrl-2088, InvivoGen).

### Western blot analysis

Mice were sacrificed after TBI for western blot analysis as previously described [41]. The solubilized proteins (5 µg per lane) were separated by sodium dodecyl sulfate‒polyacrylamide gel electrophoresis (SDS‒PAGE) and transferred to PVDF membranes (Millipore, Temecula, CA, USA). After blocking with 5% nonfat milk in Tris-buffered saline containing 0.1% Tween-20 (TBST), the membranes were incubated overnight at 4 °C with primary antibodies against the following proteins: Histone H3 (1:1000, 9715, Cell Signaling Technology), H3Cit (1: 1000, ab5103, Abcam), MPO (1:1000, ab208670, abcam), Bcl-2 (1:1000, 3498, Cell Signaling Technology), Bax (1:1000, 2772, Cell Signaling Technology), CHOP (1:1000, 2895, Cell Signaling Technology), cleaved PARP1 (1:1000, ab32064, Abcam), XBP-1s (1:1000, 12787, Cell Signaling Technology), IRE1α (1:1000, 3294, Cell Signaling Technology), phospho-IRE1α (1:300, 48187, Abcam), PAD4 (1:1000, 214810, Abcam), cleaved Caspase-3 (1:1000, 31A1067, Santa Cruz), GRP78 (1:1000, ab21685, Abcam) and β-actin (1:5000, TA-09, ZSGB-Bio).

Subsequently, the membranes were washed with TBST and incubated with appropriate secondary antibodies (1:5000, ZB-2301/ZB-2305, ZSGB-Bio) for 1 h at RT. The immunoblot bands were tagged with a Chemiluminescent HRP Substrate (EMD Millipore Corporation, USA) and visualized under an imaging system (Bio-Rad, Hercules, CA, USA). The gray values were quantified by the ImageJ program. β-Actin was used as the loading control in all immunoblots.

### Nissl staining

Nissl staining was used to estimate neuronal damage as previously described [44]. The damaged neurons were distinguished by shrunken cytoplasm along with condensed staining, while normal neurons were characterized by a relatively large and full soma.

### Immunofluorescence

Mice were euthanized under anesthesia and analgesia as described in the previous section and immediately perfused with phosphate-buffered saline (PBS; pH 7.4) via cardiac puncture followed by 4% paraformaldehyde. The brain was dissected and then embedded in OCT medium (Sakura, Oakland, CA). Coronal sections (8 µm thickness) were cut using a cryostat at -20 °C and mounted onto poly-L-lysine-coated slides. The brain cryosections were washed with PBS and permeabilized with 0.1% Triton X-100 (Sigma Aldrich) for 20 min and then 3% bovine serum albumin (BSA) for 1 h at RT. The brain sections were subsequently incubated overnight at 4 °C with primary antibodies against proteins including NeuN (1:500, ab104224, Abcam), Iba-1 (1:500, ab178847, Abcam), GFAP (1:500, 3670 S, Cell Signaling Technology), TLR9 (1:50, 371554, Abcam), H3Cit (1: 300, ab5103, Abcam), MPO (1:400, ab208670, abcam), PAD4 (1:400, ab96758, abcam), phospho-IRE1α (1:300, 48187, Abcam), and Caspase-12 (1:300, 62484, Abcam).

Thereafter, the sections were incubated with the appropriate Alexa Fluor-conjugated immunoglobulin G (IgG; 1:1000, Invitrogen) for 1 h at RT in the dark. Finally, the nuclei were counterstained with 4’,6-diamidino-2-phenylindole (DAPI, ab96758 Abcam) and imaged using an inverted fluorescence microscope (Olympus, Japan). The data were analyzed from randomly selected microscopic fields using the ImageJ program (Version 1.46r, Bethesda, MD, USA).

### Terminal deoxynucleotidyl transferase dUTP nick-end labeling (TUNEL) assay

A TUNEL assay was used to detect the DNA fragmentation of apoptotic cells in the perilesional cortex of the mouse brain after TBI with the In Situ Cell Death Detection Kit, POD (Roche, Germany), according to the manufacturer’s instructions [45].

### Modified neurological severity scores (mNSSs)

mNSSs were used to measure neurological function, as previously reported [46]. Neurological assessments were conducted at baseline before the injury and at days 1, 3, 5, 7, and 14 postinjury.

### Rotarod test

Motor coordination was evaluated with a rotating rod that accelerated linearly from 4 to 40 rpm (Rotarod, Ugo Basile) on the 1st, 3rd, 5th, 7th, and 14th days after the CCI or sham procedure. The average time mice spent on the rotating rod for three consecutive tests was recorded. The mice were permitted to run for a maximum of 5 min with a 5 min rest period between each trial, and the latency to fall was noted for three consecutive tests. The average of the three trials was taken as the result for statistical analysis.

### Injection of adenoviruses

Recombinant PAD4 adenovirus (Adeno-PAD4; Adeno-CMV-Padi4-3*Flag-tagged, 1.26 × 1011 plaque-forming units/mL) and empty adenovirus (Adeno-CMV-3*Flag-tagged) were provided by Hanbio Biotechnology (Shanghai, China). Mice were injected with 2 µl of adenovirus stereotactically into the right cortex of the brain (coordinates: 0.2 mm posterior to bregma, 2.0 mm lateral to midline, and 1.0 mm ventral to skull surface) 24 h before CCI.

### Neutrophil isolation and in vitro NETs assay

Neutrophils were isolated from bone marrow using a Neutrophil Isolation Kit (Miltenyi Biotec) according to the manufacturer’s instructions. Freshly isolated neutrophils were suspended in RPMI-1640 (Gibco, MA) and seeded at a density of 6×10^5^ cells per mL in 48-well glass-bottomed plates. After treatment with 10 mg/mL *Klebsiella pneumoniae* lipopolysaccharide (LPS; Sigma Aldrich) for 2.5 h at 37 °C, the cells were fixed in 2% paraformaldehyde, blocked with 3% bovine serum albumin, incubated with rabbit anti-citrullinated histone H3 (Cit H3; 1:1000; Abcam) overnight at 4 °C, and then incubated with Alexa Fluor 488 donkey anti-rabbit secondary antibody for 40 min at RT. The nuclei were counterstained with 4',6-diamidino-2-phenylindole (DAPI; Abcam).

### NETs preparations

Neutrophils isolated from bone marrow were suspended in RPMI 1640 (Gibco, Waltham, MA) and seeded in 6-well plates at a density of 6 × 10^5^ cells per mL. Neutrophils were stimulated with PMA (100 nM, P1585, Sigma‒Aldrich) for 4 h at 37 °C in the presence of 5% CO_2_ [47]. After removing the medium, the wells were washed twice with PBS. NETs were collected in 15-mL tubes by vigorous agitation. After centrifugation at 300 × *g* for 5 min, whole cells and debris were removed. NETs isolates were quantified by evaluating the concentration of DNA using a Quant-iT PicoGreen dsDNA Assay kit (Invitrogen).

### HT22 cell culture and drug administration

HT22 mouse hippocampal neurons were provided by the Cell Center of the Chinese Academy of Sciences (Shanghai, China). The HT22 cells were cultivated in Dulbecco’s modified Eagle’s medium (DMEM, Gibco) supplemented with 10% fetal bovine serum (Gibco) and 1% penicillin−streptomycin mix (no. C0222, Beyotime Biotechnology). The cells were maintained in an incubator at 37 °C with 5% CO2 and plated for 24 h for adhesion, after which they were subjected to follow-up experiments.

HT22 cells were preincubated with or without ODN-2088 (10 µM) 1 h prior to passaging, and then the cells were cocultured with NETs at a concentration of 300 ng/ml.

### Statistical analysis

The data are expressed as the mean ± standard deviation (SD) and were analyzed with GraphPad Prism software (GraphPad Software, Version 8.1.2 San Diego, CA, USA). The intensity of the sham/control group in the western blot analysis was standardized. Data normality of every group except sham was tested by the Shapiro‒Wilk test. Multiple comparisons were analyzed by one-way analysis of variance followed by Tukey’s multiple comparison test. When comparing two groups, unpaired Student’s *t-*test or Welch’s *t*-test was performed. The mNSS and rotarod test were analyzed by the Kruskal–Wallis test, followed by Dunn’s multiple comparisons test. A *p*-value < 0.05 was considered to indicate statistical significance.

## Supplementary information

Supplementary Figure.1

Figure Legends of Supplementary Fig. 1

Full lengths western blots in Fig. 1

Full lengths western blots in Fig. 2

Full lengths western blots in Fig. 4

Full lengths western blots in Fig. 7

Full lengths western blots in Fig. 8

Full lengths western blots in Supplementary Figure.1

## Data Availability

The datasets supporting the conclusions of this article are included within the article. All materials used in this manuscript will be made available to researchers and are subject to confidentiality.
