# Supplementary figures and images for: Neutrophil extracellular traps aggravate neuronal endoplasmic reticulum stress and apoptosis via TLR9 after traumatic brain injury
Source: Cell Death Dis. 2023 Jun 26;14(6):374. doi: 10.1038/s41419-023-05898-7 (PMC10293297; doi:10.1038/s41419-023-05898-7)

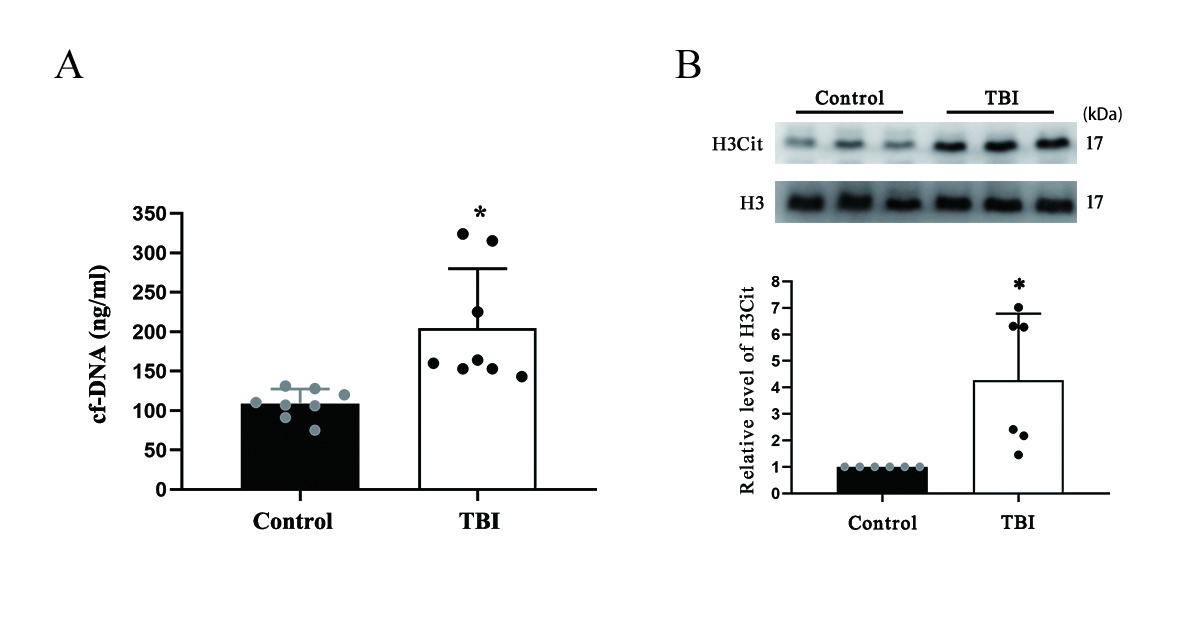

Supplement: Supplementary file 1 — Supplementary Figure.1 [file 41419_2023_5898_MOESM1_ESM.tif]

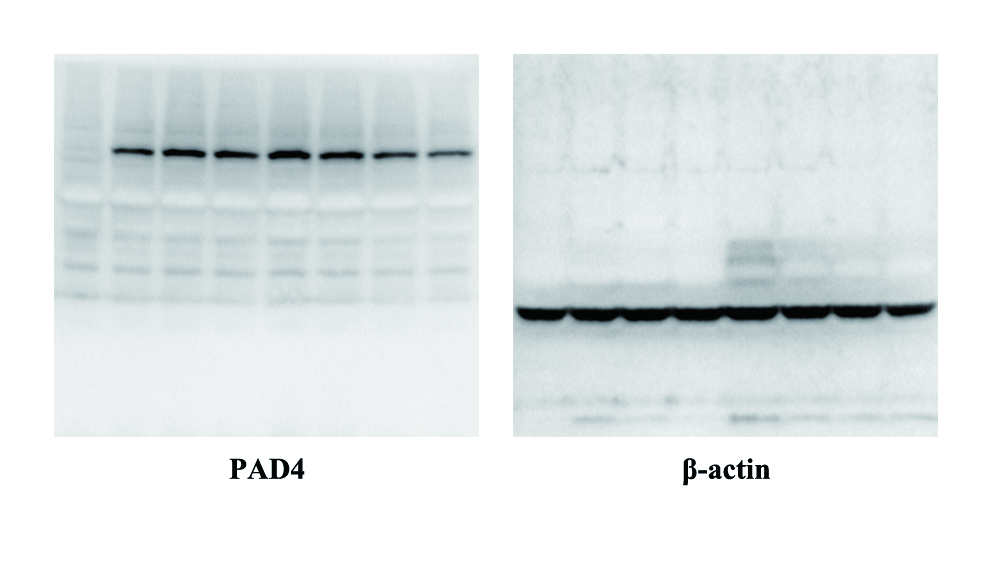

Supplement: Supplementary file 3 — Full lengths western blots in Fig. 1 [file 41419_2023_5898_MOESM3_ESM.tif]

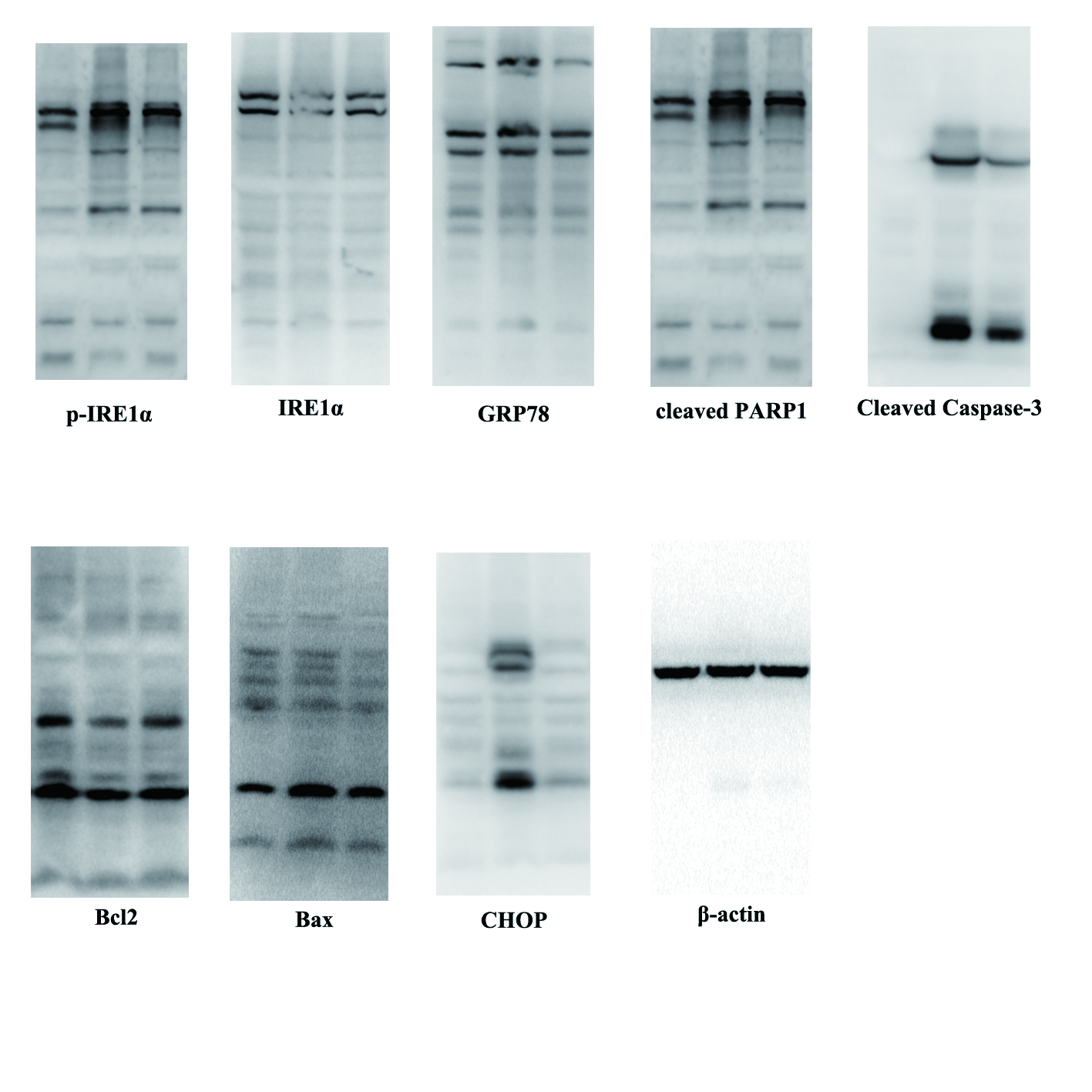

Supplement: Supplementary file 4 — Full lengths western blots in Fig. 2 [file 41419_2023_5898_MOESM4_ESM.tif]

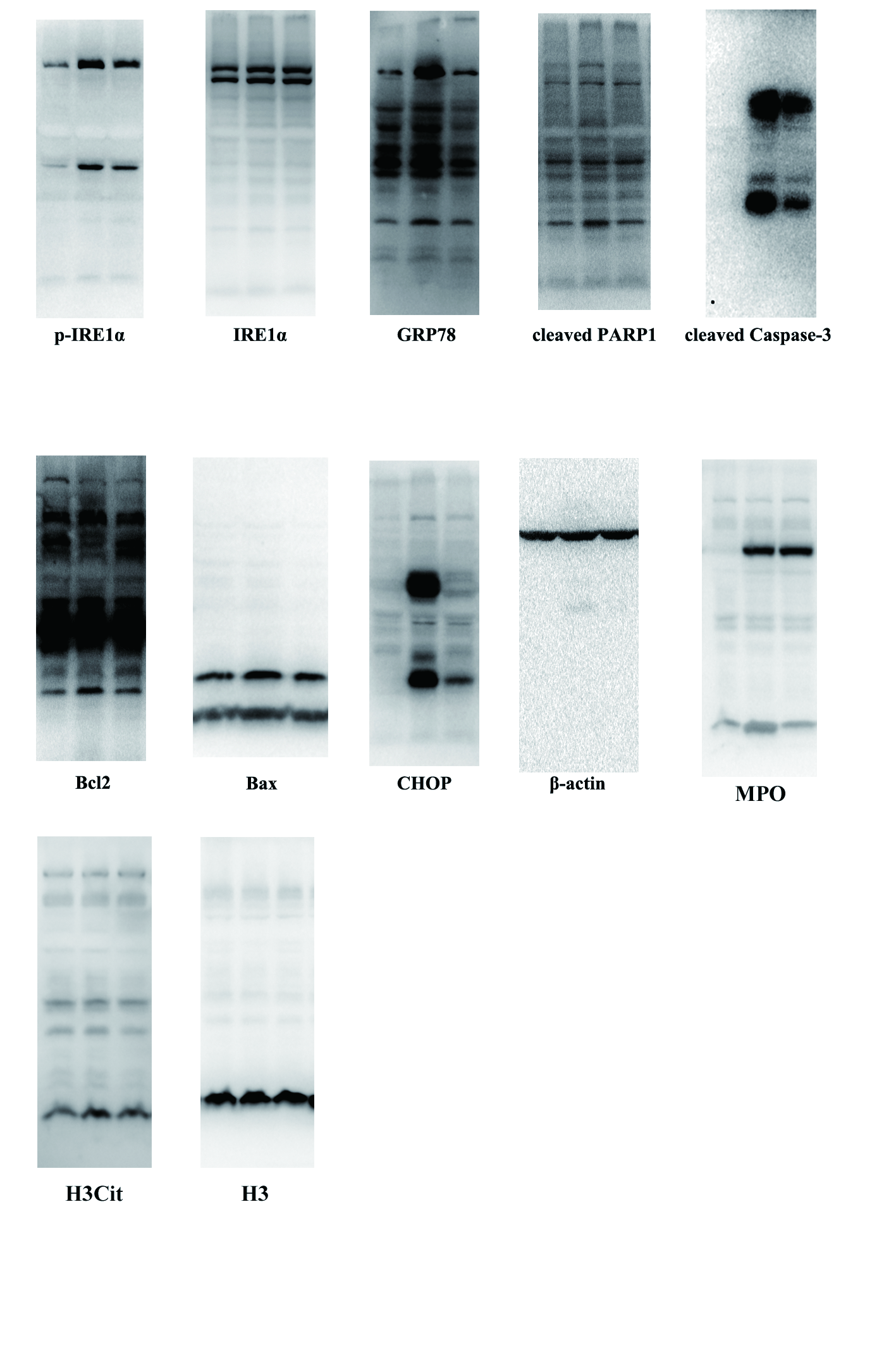

Supplement: Supplementary file 5 — Full lengths western blots in Fig. 4 [file 41419_2023_5898_MOESM5_ESM.tif]

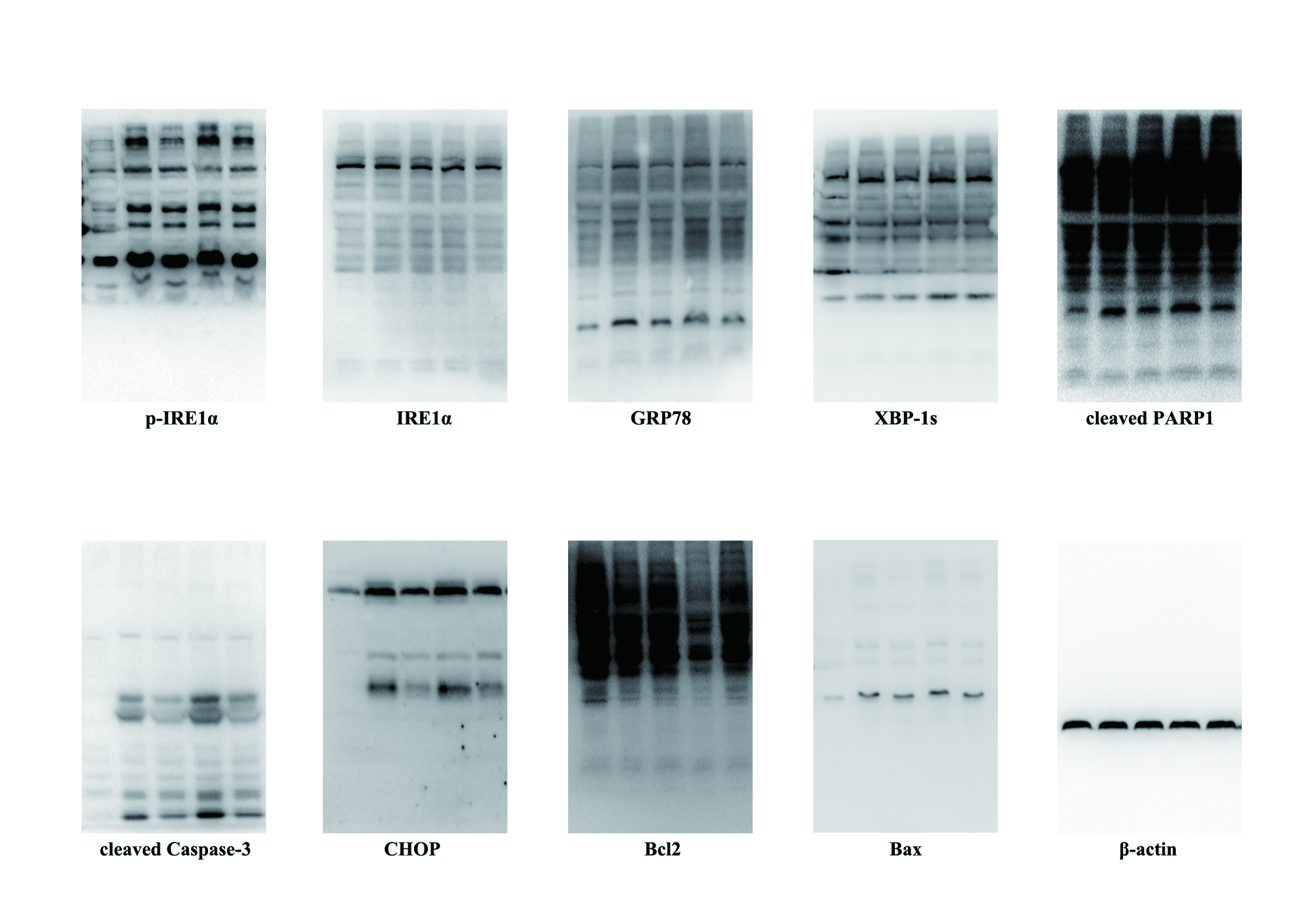

Supplement: Supplementary file 6 — Full lengths western blots in Fig. 7 [file 41419_2023_5898_MOESM6_ESM.tif]

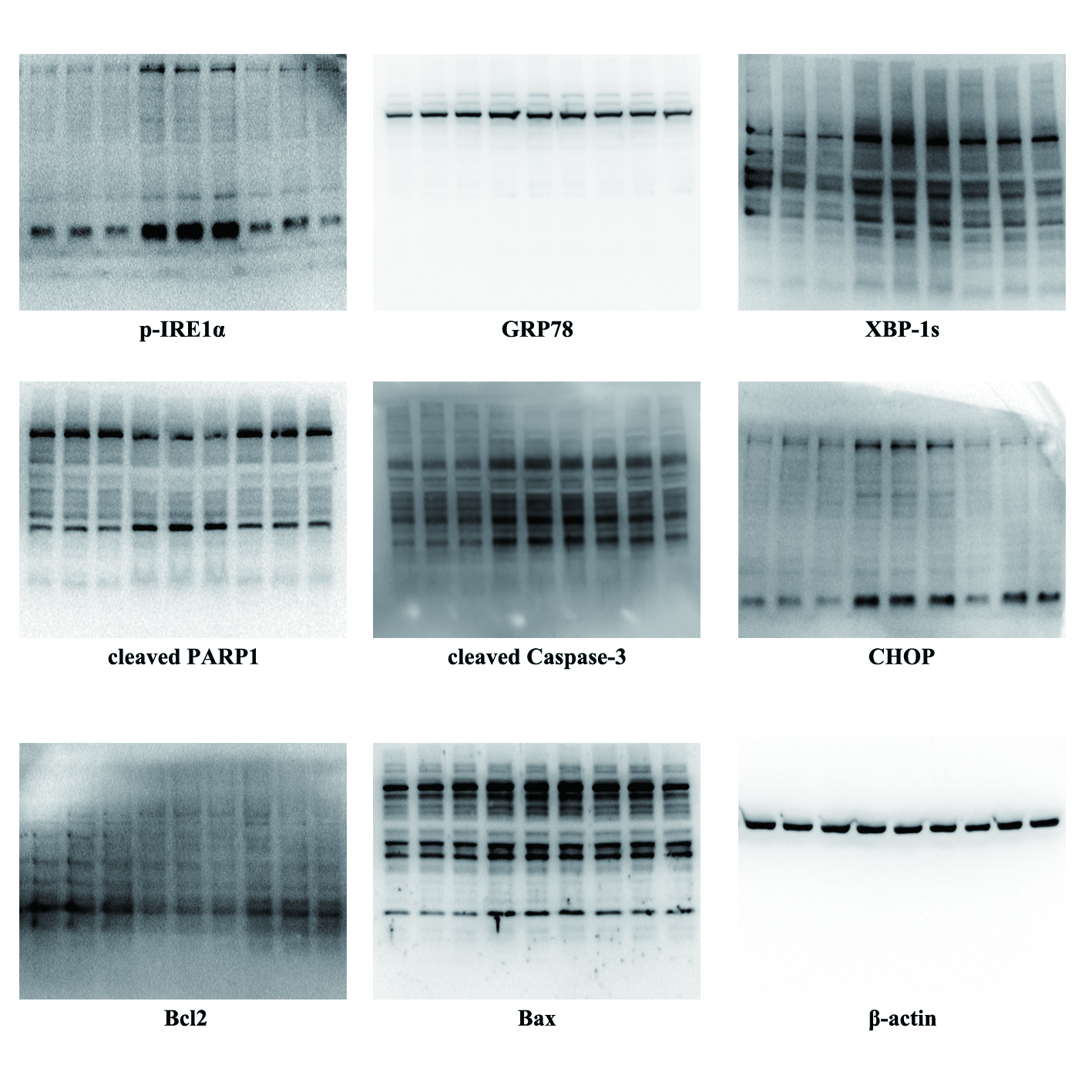

Supplement: Supplementary file 7 — Full lengths western blots in Fig. 8 [file 41419_2023_5898_MOESM7_ESM.tif]

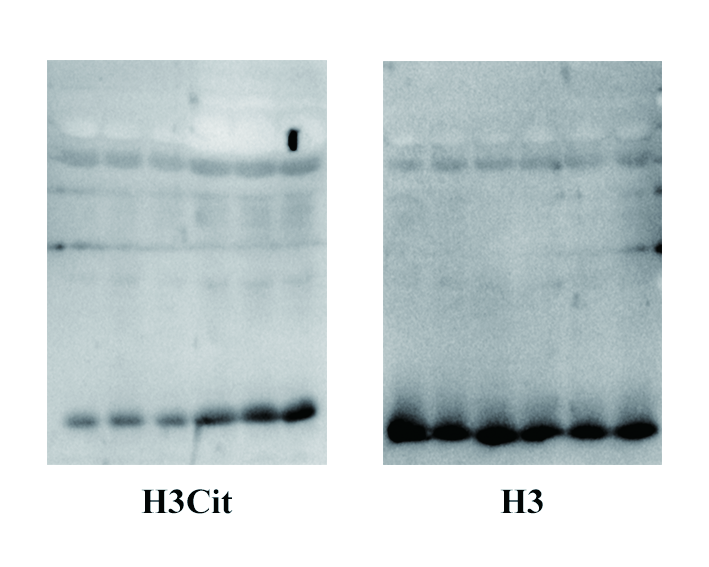

Supplement: Supplementary file 8 — Full lengths western blots in Supplementary Figure.1 [file 41419_2023_5898_MOESM8_ESM.tif]
