# Supplementary material for: Neutrophil extracellular traps aggravate neuronal endoplasmic reticulum stress and apoptosis via TLR9 after traumatic brain injury
Source: Cell Death Dis. 2023 Jun 26;14(6):374. doi: 10.1038/s41419-023-05898-7 (PMC10293297; doi:10.1038/s41419-023-05898-7)
Supplement: Supplementary file 2 — Figure Legends of Supplementary Fig. 1 [file 41419_2023_5898_MOESM2_ESM.docx]

**Supplementary Fig. 1. Plasma NET levels were increased in TBI patients. A** Quantification of extracellular DNA levels in the plasma of TBI patients and healthy controls was analyzed by a Quant-iT PicoGreen dsDNA Assay kit (Invitrogen) (n=8). **B** Protein levels of H3Cit (NETs biomarker) in the blood in the neutrophils from blood of TBI patients and controls were assessed by western blotting. The intensity was quantified using ImageJ software (n=6). NETs neutrophil extracellular traps. ER endoplasmic reticulum. TBI traumatic brain injury, H3Cit citrullinated histone 3. The data are expressed as the mean ± SD. *p < 0.05 compared with control.
